# Monitoring of Adult Zebrafish Heart Regeneration Using High-Frequency Ultrasound Spectral Doppler and Nakagami Imaging

**DOI:** 10.3390/s19194094

**Published:** 2019-09-22

**Authors:** Sunmi Yeo, Changhan Yoon, Ching-Ling Lien, Tai-Kyong Song, K. Kirk Shung

**Affiliations:** 1 Department of Electronic Engineering, Sogang University, Seoul 04107, Korea; yeosunmi@gmail.com; 2 Department of Biomedical Engineering, Inje University, Gimhae 50834, Korea; 3 Saban Research Institute, Children’s Hospital Los Angeles, Los Angeles, CA 90027, USA; clien@chla.usc.edu; 4 Department of Biomedical Engineering, University of Southern California, Los Angeles, CA 90089, USA; kkshung@usc.edu

**Keywords:** high-frequency ultrasound, Nakagami imaging, spectral Doppler, zebrafish

## Abstract

This paper reports the feasibility of Nakagami imaging in monitoring the regeneration process of zebrafish hearts in a noninvasive manner. In addition, spectral Doppler waveforms that are typically used to access the diastolic function were measured to validate the performance of Nakagami imaging. A 30-MHz high-frequency ultrasound array transducer was used to acquire backscattered echo signal for spectral Doppler and Nakagami imaging. The performances of both methods were validated with flow and tissue-mimicking phantom experiments. For in vivo experiments, both spectral Doppler and Nakagami imaging were simultaneously obtained from adult zebrafish with amputated hearts. Longitudinal measurements were performed for five zebrafish. From the experiments, the *E*/*A* ratio measured using spectral Doppler imaging increased at 3 days post-amputation (3 dpa) and then decreased to the value before amputation, which were consistent with previous studies. Similar results were obtained from the Nakagami imaging where the Nakagami parameter value increased at 3 dpa and decreased to its original value. These results suggested that the Nakagami and spectral Doppler imaging would be useful techniques in monitoring the regeneration of heart or tissues.

## 1. Introduction

The zebrafish is one of the most important models for developmental and regenerative biology, especially for cardiovascular research [1,2]. The heart of an adult zebrafish is simple (two-chambered with a single atrium and ventricle) and small (≈1 mm^3^), but its histological composition is similar to that of higher vertebrates. It has been reported that the zebrafish heart regenerates fully after amputation of up to 20% of the ventricle. Optical imaging methods are often used to assess the cardiac function and visualize the process of heart regeneration for the zebrafish embryo because of their transparency [3]. However, it is difficult to apply these technologies to the adult zebrafish because their skin becomes opaque. Thus, it is required to scarify or genetically modify the adult zebrafish to visualize the heart, making follow-up study impossible [4,5]. Other imaging modalities, such as micro-magnetic resonance imaging, micro-computed tomography, and high-frequency ultrasound imaging, have been proposed to image the anatomy of adult zebrafish hearts [6,7]. 

Among them, high-frequency ultrasound imaging capable of real-time imaging with a high spatial resolution has recently been explored and has shown promising results in the imaging of adult zebrafish. The zebrafish liver tumors were successfully characterized, and its progression and response to treatment were analyzed using 50-MHz high-frequency ultrasound imaging [8]. A mechanical sector scanning method using single-element transducers (e.g., 35, 40, and 70 MHz) was used to provide the high-resolution imaging of zebrafish hearts and blood flow velocities [9,10,11]. Recently, a dual-pulsed wave Doppler imaging method that acquired both spectral and tissue Doppler waveforms simultaneously was proposed to monitor the functional change during the regeneration of adult zebrafish [12]. This method employed a high-frequency linear array transducer whose center frequency was 30 MHz and could diagnosis the diastolic dysfunction.

In addition to the anatomical and functional analysis of zebrafish hearts using conventional ultrasound B-mode and Doppler imaging, ultrasound tissue characterization methods may be used to characterize the heart regeneration of zebrafish. In the tissue characterization methods, the statistical properties of the ultrasound backscattered signal that is related to microstructures of tissue is analyzed [13,14,15,16]. Due to the randomness of backscattering, a probability distribution was adopted to model the statistics of the backscattered signal. Among various models, the Nakagami distribution, originally used to describe the statistics of radar signal, has received attention because of its simplicity and computational efficiency [17,18,19]. It was reported that the amplitude of the received echo signal from tissues follows the Nakagami distribution, and the shape parameter of the Nakagami distribution (i.e., Nakagami parameter *m*) shows various scattering conditions including pre-Rayleigh, Rayleigh, and post-Rayleigh distributions. Since the Nakagami parameter is only dependent on the shape of the envelope distribution, it can differentiate different scatterer concentrations in a medium. In addition, the performance of Nakagami imaging for tissue characterization is less affected by different scanner settings. Nakagami imaging has demonstrated its capability for characterizing tissue properties and has shown promising results in many applications, such as cancer diagnosis, staging of liver fibrosis, and thermal ablation monitoring [20,21,22,23,24,25,26,27]. Although the Nakagami imaging has been used for many applications, it has not yet been applied to a tissue regeneration study.

In this paper, we report the feasibility of Nakagami imaging in the monitoring of adult zebrafish heart regeneration. In addition, the spectral Doppler imaging that has already been investigated to study the zebrafish ventricular function was also measured to validate the performance of the Nakagami imaging. A custom-built, 30-MHz, high-frequency array transducer and a high-frequency ultrasound imaging system were used to acquire both Nakagami and spectral Doppler imaging. The performances of these methods were validated using flow and tissue-mimicking phantoms. For in vivo experiments, adult zebrafish with amputated hearts were prepared, and longitudinal studies (one week prior to ventricular amputation and 3, 7, 14, 21, and 32 days post-amputation) were performed to monitor the heart regeneration. 

## 2. Method

### 2.1. Pulsed Wave Spectral Doppler

Pulsed wave spectral Doppler imaging provides a graphic display of velocities over time and is being used in both pre-clinical and clinical studies to estimate average blood velocity within a range gate. Doppler ultrasound uses the phase change of the received echoes (i.e., Doppler shift) from red blood cells. In the pulsed wave spectral Doppler mode, the received echo signals are demodulated to obtain inphase (*I*) and quadrature (*Q*) signals, and are filtered using highpass filters to remove clutters that originate from tissues. The spectrum of filtered *I*/*Q* signals are obtained using a Fourier transform and the Doppler shifts are converted to velocities using [12]:

(1) $$v=\frac{f_{d}}{2f_{0}cos\theta }c$$

where *f_d_* is the measured Doppler shift, *f*_0_ is the center frequency of the transducer, *θ* is the angle between the ultrasound beam and flow direction, and *c* is the speed of sound. 

### 2.2. Nakagami Imaging

The probability distribution of the ultrasound backscattered envelope, *r*, under the Nakagami model is expressed as [17,19]:

(2) $$f\left(r\right)=\frac{2m^{m}r^{2m-1}}{\Gamma \left(m\right)\Omega^{m}}\exp\left(-\frac{m}{\Omega }r^{2}\right)U\left(r\right)$$

where *Γ*(·) and *U*(·) are the gamma and the unit step functions, and *Ω* and *m* are the scaling and Nakagami parameters, respectively. The scaling and Nakagami parameters can be obtained using:

(3) $$\Omega =E\left(r^{2}\right)$$

(4) $$m=\frac{{\left[E\left(R^{2}\right)\right]}^{2}}{E{\left[R^{2}-E\left(R^{2}\right)\right]}^{2}}$$

where *E*(·) represents the statistical mean operator. The Nakagami parameter *m* is a shape parameter that allows for the classification of the envelope statistics. For the value of 0 < *m* < 1, the backscattered envelope statistics follows the pre-Rayleigh distribution. If *m* equals 1, this represents a Rayleigh distribution, and the statistics follow the post-Rayleigh distribution when *m* is larger than 1. This property allows for the Nakagami distribution to characterize the tissue compositions. Using the Nakagami parameter *m*, we can construct the Nakagami image by using a square sliding window within a region-of-interest ROI to analyze the envelope statistics with the sliding window moving throughout the entire ROI in steps of one pixel. The size of the sliding window used to estimate the Nakagami parameter was suggested to be a square with a length equal to three times the transmitted pulse length [28].

### 2.3. Experimental Setup and Evaluation Metric 

To access the feasibility of the method, we used a custom-built, 64-channel high-frequency array imaging system and a high-frequency linear array transducer with 256 elements [29]. The transducer had a 50-µm azimuth pitch (i.e., *λ* in water) and a 2-mm elevation length focused at 7.3 mm. The center frequency was 28 MHz and the −6-dB fractional bandwidth was 40%. The transducer was driven by the high-frequency ultrasound imaging system with a transmission voltage of 40 V_pp_. The transmission focal depth was 6.4 mm. The pulse repetition frequency was set to be 9.5 kHz for both phantom and in vivo experiments. 

The phantom experiments were conducted to evaluate the performances of the implemented spectral Doppler and Nakagami imaging. We used a flow phantom with a polyimide tube and a syringe pump (NE-1000 Multi-Phaser, New Era Pump System Inc., Farmingdale, NY, USA) to measure the flow velocity using a spectral Doppler ultrasound. The tube had an inner diameter of 510 µm. A blood-mimicking fluid was used and the flow velocities were controlled using the syringe pump. The Doppler angle was 61° (i.e., *θ* = 61° in Equation (1)) and three velocities—3, 5, and 10 cm/s—were used in the flow phantom experiments. The average velocity (*v*) generated by the syringe pump was computed using the flow rate (*Q*) and the radius of the tube (*r*); *v* = *Q*/(*πr*^2^). In addition, the performance of the Nakagami imaging was evaluated using conventional agar-based tissue-mimicking phantoms. The phantoms were prepared according to the method reported previously [30]. Polyamide microspheres were used as acoustic scatterers. The fabricated phantoms had identical scatterer size distributions (i.e., ≈1–10 µm) but had different scatterer concentrations of 3, 6, and 10%.

**Figure 1:**
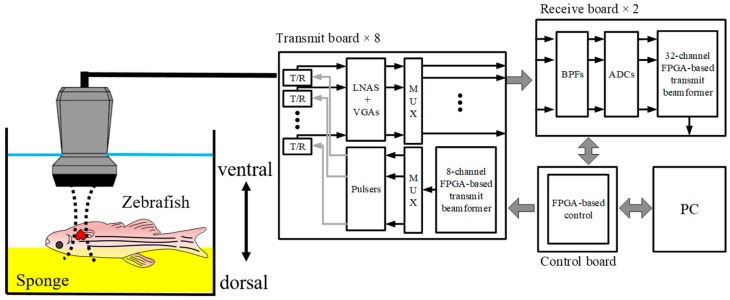
Experiment setup for adult zebrafish heart imaging. The zebrafish was anesthetized and placed on a sponge (yellow box) to position the ventral side facing upwards, and the ultrasound array transducer was positioned above.

All adult zebrafish experiments were performed in accordance with protocols approved by the Institutional Animal Care and Use Committee at the Children’s Hospital Los Angeles (protocol number 201-12). For the amputation, each zebrafish was first sedated in 5% tricaine solution (MS-222, Sigma-Aldrich, St. Louis, MO, USA). We used tweezers to remove the scale layer and then excised approximately 20% of the ventricle using scissors. Adult zebrafish experiments were performed one week prior to ventricular amputation and 3, 7, 14, 21, and 32 days post-amputation (dpa) to monitor functional changes of the heart during the regeneration process from five zebrafish whose mean size (length) was 40.8 mm. To perform the imaging, we anesthetized the zebrafish by submerging them in 0.08% tricaine solution for 30 s and then removed fish scales around the heart. The zebrafish were then fixed upside down on a sponge to position the ventral side facing upwards, as shown in Figure 1. The chamber was filled with 0.04% tricaine solution to sedate the fish during experiments. To measure blood flow, we located a range gate at the entrance of the bulbus arteriosus in between the ventricular outflow tract and the atrioventricular valve under the guidance of B-mode ultrasound imaging. We positioned the zebrafish to make the direction of atrioventricular blood flow perpendicular to the transducer, thus the angle between the ultrasound beam and flow direction was zero, i.e., *θ* = 0° in Equation (1). The beamformed radio-frequency (RF) data of B-mode and the spectral Doppler signal were acquired and then transferred to a personal computer (PC). We performed offline processing to generate spectral Doppler and Nakagami imaging using a custom-designed Matlab software (R2018b, Mathworks Inc., Natick, MA, USA).

To quantitatively evaluate the method, we measured the *E*/*A* ratio that represents the ratio of the peak velocity of blood flow in the early diastole (*E*-flow) to the peak velocity in the late diastole via atrial contraction (*A*-flow). The *E*/*A* ratio has been used to assess blood flow circulation and examine human hearts to identify diastolic dysfunction [31]. In addition, the shape parameters (i.e., Nakagami parameter *m*) were measured in a ROI located at the ventricle during the diastolic phase. The mean and standard deviation were measured from five zebrafish. The size of the sliding window for estimating the Nakagami parameter was set to be three times the pulse length (i.e., 300 µm × 300 µm), as suggested by the previous study [28].

## 3. Results and Discussion

### 3.1. Phantom Experiments

**Figure 2:**
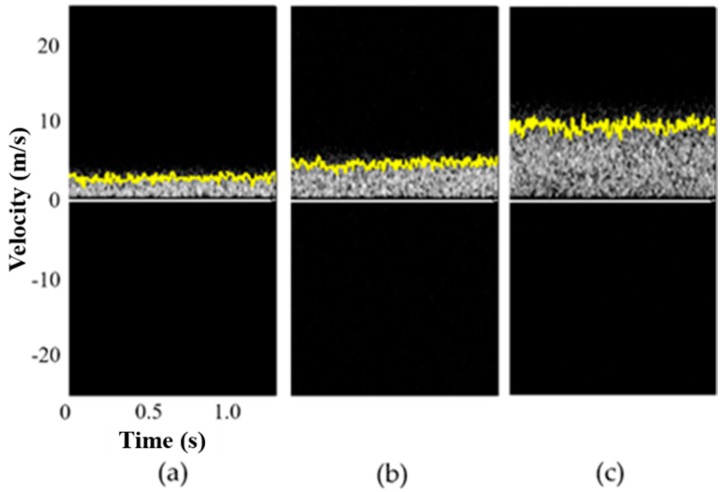
Measured flow velocities using pulsed wave spectral Doppler imaging acquired from the flow phantom with velocities of (**a**) 3, (**b**) 5, and (**c**) 10 cm/s. The measured average velocities for each case were, respectively, 2.9 ± 0.4, 4.9 ± 0.5, and 9.9 ± 0.7 cm/s.

Figure 2 shows the pulsed spectral Doppler waveforms acquired from the flow phantom with velocities of 3, 5, and 10 cm/s. The yellow lines represent the velocity envelopes. The measured average velocities for each case were, respectively, 2.9 ± 0.4, 4.9 ± 0.5, and 9.9 ± 0.7 cm/s, which agreed well with flow velocities produced by the syringe pump. Note that the direction of flow was toward the transducer, thus positive velocities were obtained. If the flow direction was away from the transducer, negative velocities would have been observed. 

**Figure 3:**
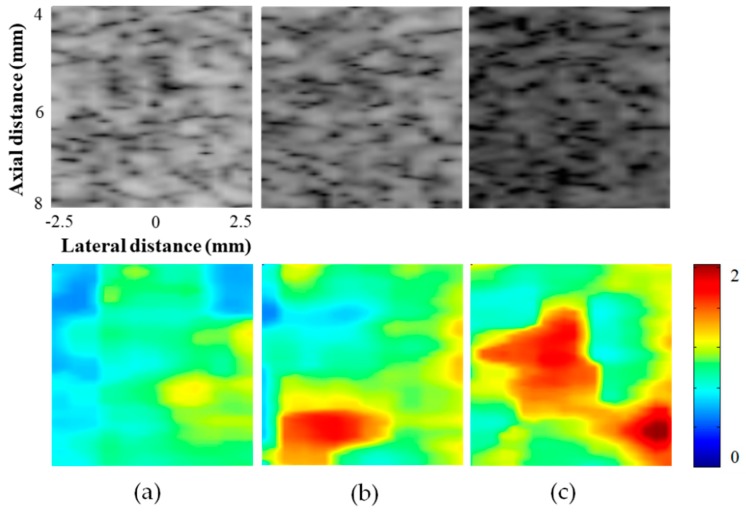
Ultrasound B-mode images and corresponding Nakagami images from the tissue-mimicking phantoms with different scatterer concentrations of (**a**) 3, (**b**) 6, and (**c**) 10 %. The phantoms with higher scatterer concentrations yielded higher Nakagami parameter values.

B-mode images with corresponding Nakagami images from the tissue-mimicking phantoms with different scatterer concentrations of 3, 5, and 10 % are shown in Figure 3. As shown in Figure 3, the intensity of the B-mode image with a higher concentration produced a lower magnitude of backscattered echoes. This was because the ultrasound attenuation increased as the concentration of scatterers increased [30]. However, the Nakagami parameter *m*, which is independent from the magnitude of echoes, yielded higher values as the scatterer concentrations increased. The mean Nakagami parameter *m* values were 0.89, 1.10, and 1.20 for the phantoms with scatterer concentrations of 3, 5, and 10 %, respectively, suggesting that distributions of echo amplitudes changed from a pre-Rayleigh to a post-Rayleigh distribution as the concentration of scatterers increased. These results are consistent with the results reported previously [19]. Note that the speckle’s signal-to-noise ratios (SSNRs), which is defined as the ratio of the mean intensity to the standard deviation in the speckle region, for each phantom were 1.88, 1.85, and 1.86, respectively, indicating that the speckle patterns of fabricated phantoms were fully developed [32].

### 3.2. In Vivo Experiments

**Figure 4:**
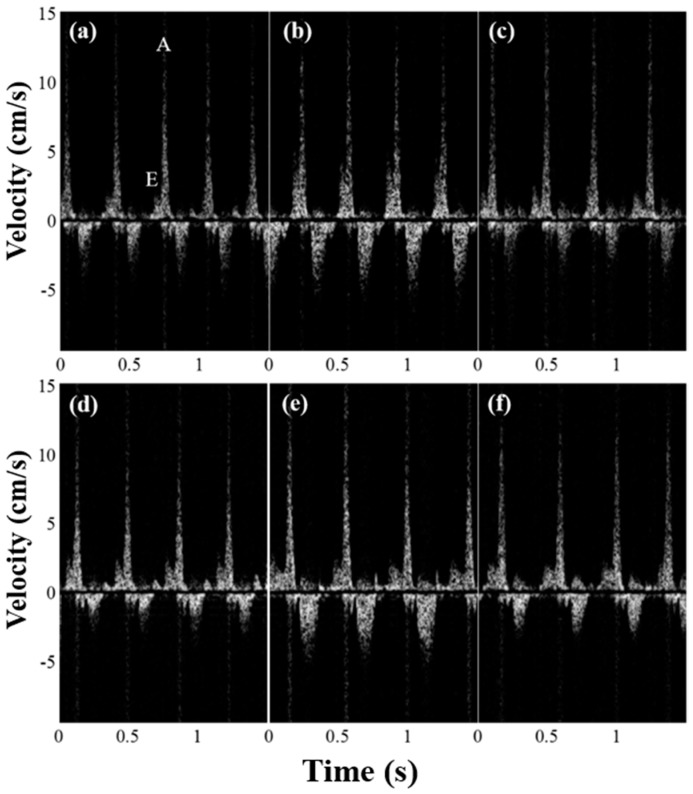
Spectral Doppler waveforms acquired from the adult zebrafish (**a**) one week prior to ventricular amputation and (**b**) 3, (**c**) 7, (**d**) 14, (**e**) 21, and (**f**) 32 days post-amputation (dpa) to monitor functional changes of the heart during regeneration. Examples of an *E*-flow and *A*-flow are indicated in Figure 4a.

**Figure 5:**
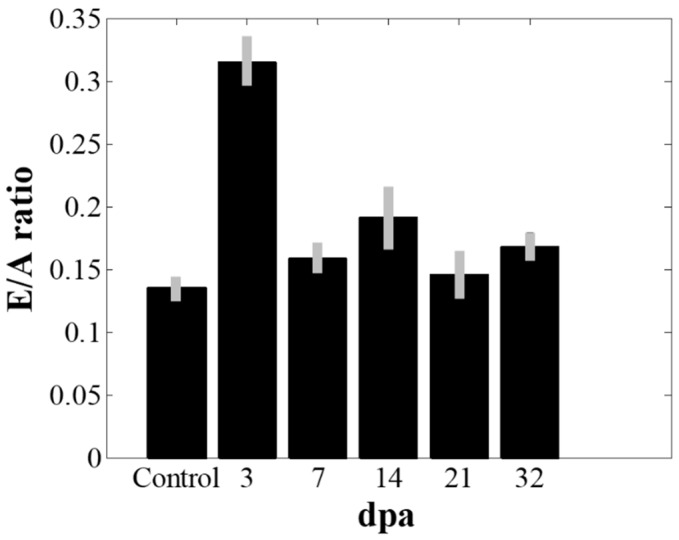
The changes of the *E*/*A* ratio values that represents the ratio of the peak velocity of blood flow in the early diastole (*E*-flow) to the peak velocity in the late diastole via atrial contraction (*A*-flow) before and after amputation. The *E*/*A* value increased at 3 dpa (*p*-value < 0.05) and recovered to the value before amputation. The error bars indicate the standard deviation in the data from five zebrafish.

Figure 4 shows the spectral Doppler waveforms at the entrance of the bulbus arteriosus from the amputated zebrafish hearts under the guidance of B-mode imaging. The *E*- and *A*-flows are indicated in Figure 4a. As shown in Figure 4, the velocity of the *E*-flow that represents the early passive filling of the ventricle increased after amputation and gradually almost returned to the values before amputation, indicating the diastolic dysfunction had recovered within a few weeks. The mean velocities of *E*-flow were 1.9 and 2.9 mm/s (*p*-value < 0.05) before amputation and 3 days post- amputation (3 dpa), respectively. However, there were no noticeable changes in *A*-flow after amputation (*p*-value > 0.05). The *E*/*A* ratios from the measured *E*- and *A*-flows are shown in Figure 5. Error bars indicate the standard deviations in the data from the five zebrafish. The increase of the *E*/*A* ratio was observed at 3 dpa (*p*-value < 0.05) and its value almost returned to the value before amputation (i.e., control) at 7 dpa. These results are consistent with the previous study [12]. From the previous study, it was reported that an increase of the *E*/*A* ratio is caused by the increased pressure gradient between the heart chambers [12]. From the previous study, it was reported that the tissue motion at the atrioventricular valve was reduced after amputation, indicating that the cardiac function might be reduced. These changes in the amputated zebrafish hearts were similar to the restrictive filling pattern observed in human hearts suffering from diastolic dysfunction caused by a reduction in ventricular compliance. Note that the range of *E*/*A* ratio values from zebrafish are different from those of humans.

**Figure 6:**
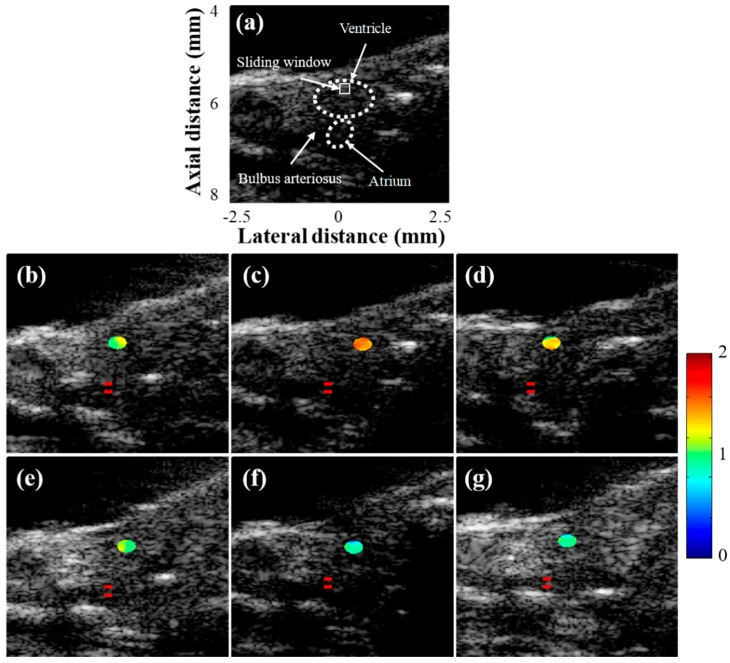
(**a**) B-mode image acquired from the adult zebrafish and Nakagami images overlaid on B-mode images from the zebrafish (**b**) one week prior to ventricular amputation, (**c**) 3, (**d**) 7, (**e**) 14, (**f**) 21 and (**g**) 32 days post-amputation (dpa). The Nakagami image was only constructed at the amputated regions. The location of Doppler gates in each image are indicated with red lines.

**Figure 7:**
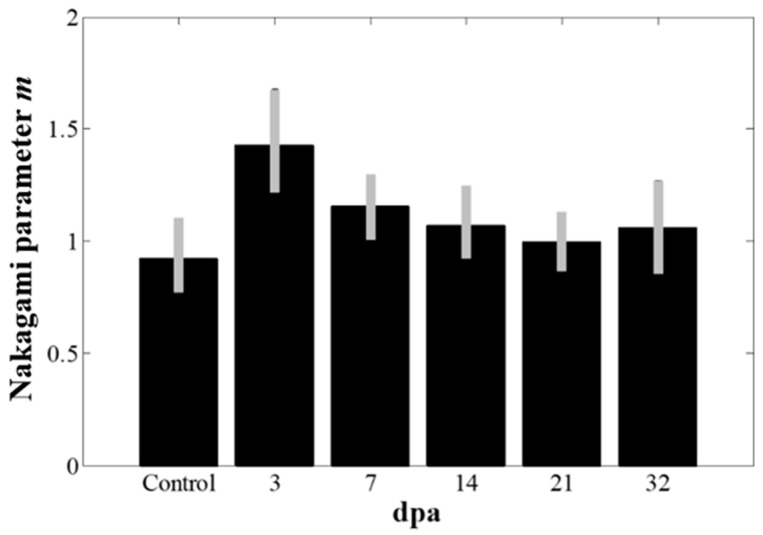
The changes of the Nakagami parameter *m* values before and after amputation. The Nakagami parameter value significantly increased at 3 dpa (*p*-value < 0.05) and recovered to the value before amputation. The error bars indicate the standard deviation in the data from five zebrafish.

Figure 6 shows the Nakagami images overlaid on the B-mode images from the same zebrafish used in the spectral Doppler experiments. The Nakagami images were constructed only for the amputated regions. The mean Nakagami parameter values in the ROIs with the standard deviation from the five zebrafish were measured and plotted in Figure 7. As shown in Figure 7, the Nakagami parameter value increased at 3 dpa (*p*-value < 0.05) and then decreased to the value before amputation. From the results, the Nakagami parameter value before amputation was 0.9, indicating the statistics of the envelope followed the pre-Rayleigh distribution. However, the statistics of the echo conformed to the post-Rayleigh distribution at 3 dpa where the mean Nakagami parameter value was 1.4, and returned to the Rayleigh distribution (the mean Nakagami parameters were 1.2, 1.1, 1.0, and 1.1 at 7, 14, 21, and 32 dpa, respectively) during heart regeneration. After the heart was amputated, fibrin clots are formed to block the hemorrhage and damaged myocardium is regenerated [2]. From the previous study, increased echo intensity was observed from the fibrin clot formations in amputated zebrafish, which could be visualized using high-frequency ultrasound B-mode imaging [10]. During the clot formation, a fibrin mesh was formed and red blood cells (RBCs) were trapped in the network. These trapped RBCs were acting as acoustic scatterers and would increase the Nakagami parameter, as well as the echo intensity. From the longitudinal studies, the changes of the Nakagami parameter values could be an indicator of heart recovery, although statistical analysis using a large number of zebrafish should be undertaken to ascertain the effectiveness of the methods.

## 4. Conclusion

In this paper, we demonstrated that Nakagami imaging could be used to monitor the heart regeneration of adult zebrafish. In addition, the spectral Doppler waveforms that have been proved to be a useful technique to study the zebrafish ventricular function were also measured to show the effectiveness of the Nakagami imaging. In the longitudinal studies of zebrafish heart regeneration, we could observe the changes of both the Nakagami parameter *m* values and spectral Doppler waveforms (i.e., *E*/*A* ratio) and diagnose the diastolic dysfunction of restrictive filling noninvasively. The amputated hearts were mostly recovered within a few weeks, which was in good agreement with previous studies. These results suggest that the Nakagami imaging, along with spectral Doppler imaging, can be used to monitor the heart and tissue regeneration process.
